# Precision perioperative AI: from signals, images, and records to applications in anesthesia-a narrative mini-review proposing an operational framework

**DOI:** 10.3389/fmed.2026.1811197

**Published:** 2026-04-09

**Authors:** Yoann Elmaleh, Karim Guessous, Bernard Victor Delvaux

**Affiliations:** Quincy Anesthésie, Hôpital Privé Claude Galien, Ramsay Santé, Quincy-sous-Sénart, France

**Keywords:** artificial intelligence, perioperative care, machine learning, risk stratification, decision support, intraoperative monitoring, postoperative complications, algorithmovigilance

## Abstract

Artificial intelligence (AI) is increasingly positioned as an assistive and decision-support layer across the perioperative pathway, transforming heterogeneous clinical data into patient-specific risk estimates and management recommendations. Yet perioperative AI remains conceptually fragmented: terms such as “precision,” “real-time,” and “multimodal integration” are frequently invoked without operational definition, and many studies still prioritize retrospective discrimination over calibration, workflow integration, and prospective clinical impact. This mini-review provides operational definitions for precision perioperative AI, real-time inference, and multimodal integration within the specific constraints of perioperative care, then synthesizes representative applications across the preoperative, intraoperative, and postoperative phases, emphasizing perioperative-specific evidence and implementation maturity. Preoperatively, machine-learning models trained on electronic health records and multimodal data can improve individualized risk stratification, supporting triage, shared decision-making, and tailored prehabilitation or monitoring strategies. Intraoperatively, waveform-based early-warning systems can reduce the duration and severity of hypotension when embedded in treatment protocols; reinforcement-learning approaches and closed-loop controllers are being explored for anesthetic depth and hemodynamic control. Computer vision applications include support for ultrasound-guided regional anesthesia and operating-room scene analysis. Postoperatively, AI-enhanced surveillance combines continuous monitoring with perioperative risk profiles to detect deterioration and forecast complications such as sepsis, acute kidney injury, and delirium. We argue that perioperative AI must be evaluated as a clinical intervention rather than a static classifier. Deployment-grade requirements include robust calibration, external validation, decision-curve analysis, human-in-the-loop design, drift detection, and structured lifecycle oversight (“algorithmovigilance”), aligned with emerging regulatory expectations and real-world perioperative workflows.

## Introduction

Perioperative medicine encompasses a uniquely dynamic segment of clinical care, spanning preoperative assessment and optimization, intraoperative anesthesia and surgery, and postoperative recovery. Across this continuum, clinicians must integrate heterogeneous data streams that differ in structure, granularity and temporal resolution. Structured electronic health record (EHR) variables, laboratory values, imaging studies, free-text documentation, medication exposures and high-frequency physiological waveforms must be synthesized in real time to support decisions that may have immediate and irreversible consequences. The cognitive and temporal demands of this environment make perioperative care a particularly relevant domain for artificial intelligence (AI) and machine learning applications (1–2).

Over the past decade, perioperative AI has evolved from retrospective proof-of-concept modeling to early clinical deployment in selected domains. Preoperatively, machine-learning models trained on large EHR datasets have demonstrated improved discrimination for major complications and mortality compared with traditional risk scores, enabling individualized risk estimation and potentially more precise triage strategies (3–5). Intraoperatively, waveform-based algorithms have been developed to predict hypotension minutes before its occurrence, and randomized trials suggest that their use can reduce the depth and duration of intraoperative hypotension when integrated with treatment protocols (2, 6–7). Parallel efforts explore reinforcement-learning–based hemodynamic control, closed-loop anesthetic systems, and context-aware ultrasound guidance (8–10). Postoperatively, AI-enhanced monitoring systems combine perioperative variables with continuous ward or ICU data to identify clinical deterioration earlier than traditional early warning scores (11–12).

Despite this rapid expansion, conceptual and methodological heterogeneity persists. Terms such as *precision perioperative AI*, *real-time*, and *multimodal integration* are widely used yet inconsistently defined. In some reports, “real-time” may refer to second-by-second waveform processing in the operating room, whereas in others it describes hourly ward-based updates. Similarly, “multimodal” may denote fusion of structured EHR data and imaging in one context, and fusion of waveforms and medication streams in another. Without operational definitions, comparisons across studies remain difficult and translation into routine practice is hindered (1–2).

In addition, much of the current literature emphasizes model discrimination, often summarized by area under the receiver operating characteristic curves, while less attention is devoted to calibration, clinical utility, behavioral impact or unintended consequences (13–14). In perioperative environments characterized by rapid transitions such as induction and emergence, small predictive gains may alter vasopressor exposure, fluid administration or sedative dosing, potentially introducing new risks even while mitigating others (15–16). Algorithmic bias, dataset shift across institutions, alarm fatigue and privacy concerns related to operating room video or high-resolution physiological data further complicate implementation (17–18).

Perioperative AI should therefore be conceptualized not merely as predictive modeling, but as a clinical intervention embedded within complex workflows. Its evaluation must extend beyond retrospective accuracy metrics to include calibration stability, human–machine interaction, downstream physiological effects, governance structures and lifecycle monitoring. The notion of algorithmovigilance, continuous post-deployment surveillance of performance and safety, may be particularly relevant in perioperative settings where practice patterns evolve rapidly and patient case mix fluctuates (17–18).

**Figure 1:**
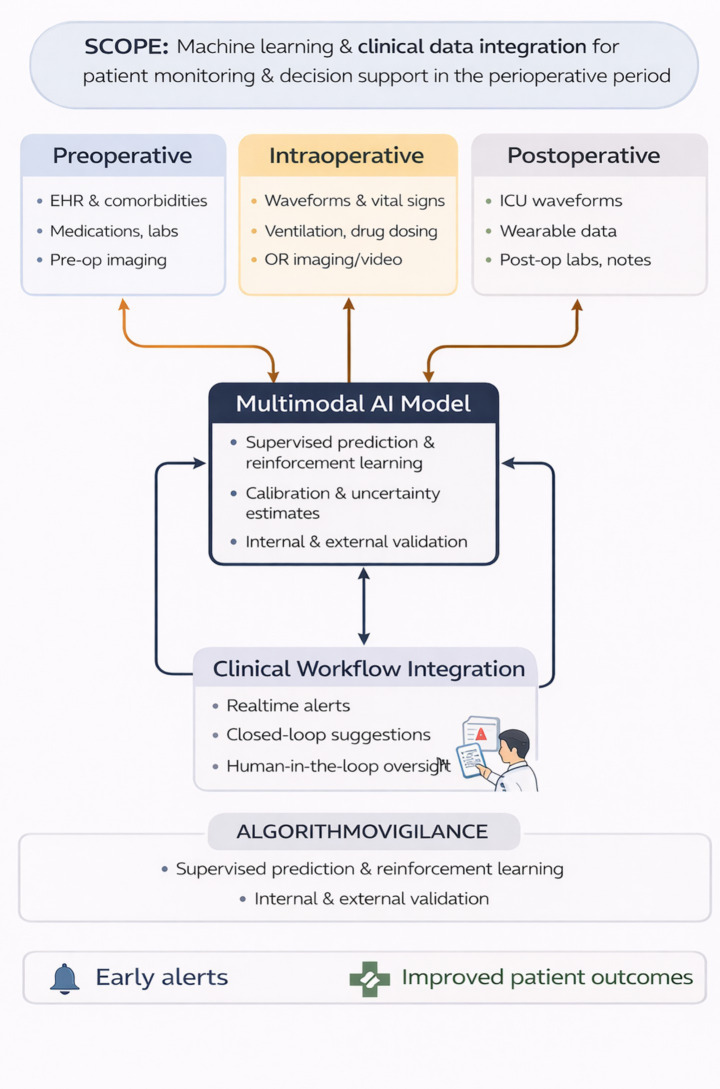
Conceptual pipeline for precision perioperative artificial intelligence. Perioperative data from the preoperative, intraoperative, and postoperative phases feed a multimodal integration layer, which produces model-based predictions. These are deployed as clinical decision support under local governance, with explicit attention to real-time constraints in the operating room and on wards/ICU.

The purpose of this mini-review is threefold. First, we propose operational definitions for precision perioperative AI, real-time inference and multimodal integration that are explicitly anchored in perioperative workflows. Second, we synthesize representative applications across the preoperative, intraoperative and postoperative phases, with careful alignment between claims and perioperative-specific evidence. Third, we examine benefit–risk trade-offs within concrete perioperative scenarios and discuss governance, validation and lifecycle monitoring requirements necessary for responsible deployment. Figure 1 presents an operational architecture of precision perioperative AI, while the accompanying tables summarize specific models, domains and levels of evidence.

By reframing perioperative AI as a workflow-embedded clinical system rather than a standalone predictive tool, this review aims to provide a structured foundation for both scientific evaluation and responsible implementation in anesthesia and perioperative medicine. Table 1 outlines key AI applications by perioperative phase, and Table 2 categorizes predictive AI use cases by data modality.

**Table 1:** Clinical applications of AI across the perioperative phases.

Perioperative phase	Selected AI applications and examples
Preoperative	Risk Stratification and Outcome Prediction: Machine learning models predict patient-specific risk of postoperative complications (e.g., mortality, cardiac events, acute kidney injury) better than traditional scores, enabling tailored surgical planning (13). Patient Evaluation and Triage: NLP and predictive algorithms auto-extract medical history and flag high-risk conditions from records; AI risk tools identify which patients may benefit from prehabilitation or ICU postoperative care. Imaging Analysis for Planning: AI interpretation of preoperative imaging (CT/MRI) assists in surgical planning (e.g., tumor characterization, anatomy mapping) and in identifying factors (airway morphology, etc.) that could complicate anesthesia (31–32). Patient Engagement: Chatbot and virtual reality systems powered by AI help educate patients and reduce anxiety by providing personalized pre-op instructions and answering FAQs (13).
Intraoperative	Real-Time Monitoring and Alerts: AI-driven early warning systems analyze physiological signals to predict events like hypotension or hypoxemia before onset, alerting clinicians for proactive management (7). AI-Guided Anesthesia Delivery: Closed-loop control systems (reinforcement learning or rule-based) adjust anesthetic drug infusion and fluids in real time to maintain stable vital signs and adequate anesthesia depth (8). Image-Guided Surgery and Navigation: Computer vision algorithms process surgical video or ultrasound images to identify anatomy and guide interventions, e.g., highlighting nerves on ultrasound for regional blocks (24) or recognizing surgical phases and instruments in laparoscopic video to assist the surgeon. Robotics and Automation: Integration of AI in robotic surgery to enable autonomous sub-tasks (like suturing) and enforce safety constraints, as well as smart dashboards that provide context-aware decision support to the surgical team.
Postoperative	Complication Prediction and Early Detection: Machine learning models continuously evaluate post-op vital signs and labs to forecast complications (sepsis, bleeding, respiratory failure) and trigger early intervention before clinical deterioration (32–35). Personalized Pain Management: Predictive models estimate analgesic needs; nociception-monitoring AI systems adjust opioid/PCA dosing to patient-specific pain responses, improving pain control while avoiding oversedation (3). Recovery and Readmission Prevention: Wearable sensors and AI telemonitoring track patients after discharge – e.g. activity, heart rate, wound images – to identify those at risk of readmission or complications (infection, VTE) and enable timely outpatient interventions.

**Table 2:** Predictive AI use cases by modality of data.

Primary data modality	Example predictive use cases in perioperative care
Physiological signals	*Intraoperative event prediction:* Real-time analysis of continuous vital signs and waveforms to forecast hypotension, tachyarrhythmias, hypoxemia, or hemorrhage, allowing preemptive management (7).
	*Sedation and nociception monitoring:* EEG, EMG, PPG, and other biosignals integrated via deep learning to gauge depth of anesthesia and pain responses, guiding anesthetic and analgesic dosing minute-by-minute (36).
	*Postoperative early warning:* Continuous ward monitoring (heart rate, respiratory rate, SpO₂) with ML-based alarm systems that detect patient deterioration (e.g., sepsis, respiratory failure) earlier than standard criteria, prompting rapid response (37).
Medical imaging	*Preoperative imaging risk assessment:* AI radiomics applied to preop scans (CT/MRI) to predict surgical complexity or likelihood of outcomes (e.g., tumor recurrence, organ injury), supplementing clinical risk models (22).
	*Intraoperative visual guidance:* Computer vision on surgical camera feeds for instrument tracking, surgical phase recognition, and identification of anatomical landmarks or pathology (tumor margins, vessels) in real time to aid surgeons (38).
	*Ultrasound-assisted procedures:* Deep learning models for ultrasound image interpretation that automatically highlight nerves, vessels, or epidural space to increase accuracy and success of regional anesthesia and line placements (26).
Clinical records (EHR)	*Outcome prediction models:* Multivariate ML models using patient history, comorbidities, labs, and operative details from EHR to predict risks of complications like AKI, surgical site infection, prolonged length of stay, or 30-day readmission (31).
	*Decision support and resource allocation:* AI algorithms analyzing EHR data to recommend clinical actions (e.g., need for ICU vs. ward, optimal timing of surgery, or specific interventions) and to optimize scheduling or resource use.
	*Documentation and workflow:* NLP-driven systems that read and summarize clinical notes or handoff reports to ensure critical information (allergies, difficult airway, etc.) is captured and communicated; also AI for coding and quality improvement from record analysis (1).

## Operational definitions and scope

This section defines three core concepts used throughout the review: precision perioperative AI, real-time perioperative AI and multimodal integration.

Precision perioperative AI refers to AI systems that deliver patient-specific predictions or recommendations intended to modify perioperative management for a particular patient. Examples include preoperative risk scores that alter triage or postoperative disposition, intraoperative early-warning systems whose alerts change hemodynamic strategy, and postoperative models that trigger specific prevention bundles for delirium or respiratory failure (3, 19–21). Generic descriptive dashboards or purely retrospective analyses that do not influence individual care are not considered here.

Real-time perioperative AI describes AI systems that update predictions or recommendations during ongoing care with a latency that still allows clinicians to intervene before clinically important harm occurs. In the operating room, real-time typically means seconds to a few minutes, based on continuous arterial pressure, electrocardiography, pulse oximetry and gas analysis (6–7). In the PACU, wards or intensive care units, real-time generally implies event-based or 5–60-min updates synchronized with vital-sign sampling and nursing assessments (12–13). A once-daily batch update of a 30-day mortality model does not meet this criterion for intraoperative control, even if it is clinically useful for longer-horizon decisions.

Multimodal integration is understood as the combination of two or more heterogeneous data modalities within a single AI system. In perioperative medicine, common multimodal combinations include structured EHR variables with intraoperative waveforms such as arterial pressure or plethysmography, EHR data with imaging such as echocardiography or computed tomography, ultrasound video with probe motion and device metadata for regional anesthesia, and EHR data with free-text notes processed through natural-language processing (1, 3, 4, 10, 22). Simply increasing the number of variables within a single modality does not constitute multimodal integration.

Figure 1 illustrates a conceptual pipeline for precision perioperative AI. Perioperative data streams from the preoperative, intraoperative and postoperative phases feed a multimodal integration and modeling layer. Outputs are deployed as real-time risk estimates, alerts or assistive control within perioperative workflows, under human oversight and continuous performance monitoring.

## Preoperative applications

Preoperative evaluation aims to quantify perioperative risk, guide optimization strategies, inform shared decision-making and determine postoperative disposition. This phase is particularly suited to AI-based approaches because it relies on heterogeneous but largely static data available before surgery. Structured EHR variables, laboratory values, medication exposures, prior admissions, imaging findings and clinical narratives collectively describe a patient’s physiological reserve and procedural risk. Machine-learning models trained on large perioperative datasets have been developed to predict composite postoperative complications, short-term mortality, acute kidney injury, cardiac events and unplanned ICU admission, frequently demonstrating improved discrimination compared with traditional rule-based scores such as ASA-PS or RCRI (1, 3, 4, 10).

However, the clinical relevance of improved discrimination must be interpreted cautiously. An increase in area under the receiver operating characteristic curve does not necessarily translate into better patient outcomes. For preoperative AI systems to influence perioperative care meaningfully, three conditions are essential: calibration accuracy across risk strata, reproducibility in external cohorts, and linkage to actionable clinical pathways (2). Poor calibration may lead to systematic overestimation of risk in low-risk patients or underestimation in high-risk individuals, potentially distorting triage decisions. External validation is particularly critical in perioperative medicine, where case mix, surgical complexity and institutional practices vary widely across centers (3).

Importantly, risk prediction must be connected to predefined management strategies. High-risk identification may trigger prehabilitation programs, optimization of anemia, tighter glycemic control, cardiology evaluation, enhanced intraoperative hemodynamic monitoring or planned postoperative HDU/ICU admission. Without such structured pathways, risk prediction remains informational rather than interventional. Decision-curve analysis and net-benefit frameworks provide tools to quantify whether model-guided decisions outperform default strategies across clinically relevant thresholds, especially when predictions influence high-cost or capacity-limited resources such as critical care beds (1).

**Figure 2:**
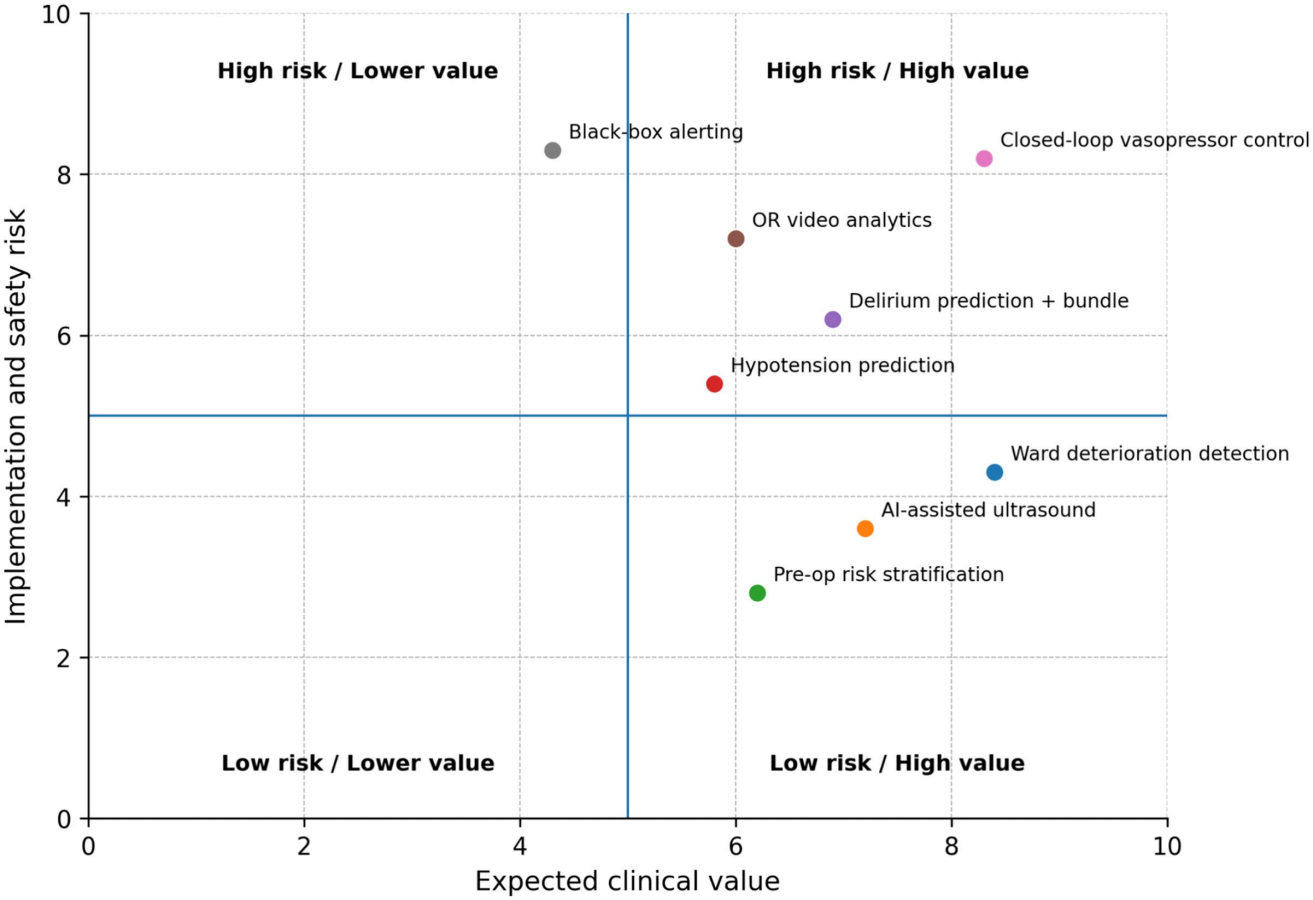
Structured risk–benefit framework for perioperative artificial intelligence applications. Applications are positioned according to their expected clinical value and their implementation and safety risk. Systems located in the lower-right quadrant may represent early deployment targets, whereas high-value but high-risk applications require strengthened governance, prospective validation, and careful integration into perioperative workflows.

Preoperative AI also enables phenotypic refinement beyond traditional risk factors. Multimodal models combining structured EHR variables with imaging-derived features have been proposed to improve prediction of organ-specific complications. For example, CT-derived skeletal muscle mass and radiographic frailty markers may add incremental predictive value for postoperative pulmonary or cardiovascular complications beyond conventional comorbidity indices (3–4). Similarly, echocardiographic parameters integrated into machine-learning pipelines may enhance cardiac risk stratification in selected surgical populations (Figure 2).

Natural-language processing (NLP) offers an additional dimension by extracting clinically meaningful information from unstructured documentation. Pre-anesthetic assessments, cardiology reports and prior operative notes frequently contain nuanced descriptions of airway difficulty, frailty, exercise tolerance or social vulnerability that are not fully captured in structured fields. NLP-enhanced models can recover these features and potentially reduce information loss inherent to structured coding systems (22). Nevertheless, NLP models are particularly vulnerable to institutional documentation habits and may not generalize across centers with different note structures.

Interpretability and transparency are critical in the preoperative setting, where predictions directly influence discussions with patients. Explainability techniques such as SHAP or related attribution methods allow clinicians to examine which variables contribute most strongly to a given prediction (23). In the context of shared decision-making, this is especially important. A model that identifies advanced age, renal impairment and reduced ejection fraction as dominant contributors is intuitively interpretable and clinically coherent. Conversely, reliance on surrogate variables such as specific order sets or documentation artifacts may signal information leakage or embedded practice bias.

Finally, preoperative models are susceptible to confounding by treatment intention and practice patterns. For instance, institutional policies regarding ICU admission or surgical selection can become encoded in the training data, leading the model to reproduce institutional behavior rather than purely patient-specific biological risk. Temporal dataset shift is also relevant: evolving perioperative protocols, enhanced recovery pathways and changing case complexity can degrade model performance over time. Continuous monitoring and periodic recalibration are therefore necessary to preserve accuracy and avoid drift-related misclassification (17–18).

In summary, preoperative AI offers substantial promise for individualized risk stratification and resource allocation. Its clinical value depends not only on statistical performance but also on calibration robustness, external validity, integration with predefined optimization pathways, interpretability for patient communication and longitudinal performance monitoring within dynamic perioperative systems.

## Intraoperative applications

The intraoperative period is characterized by rapid physiological fluctuations, pharmacodynamic variability and continuous high-density monitoring. Unlike the preoperative phase, where data are largely static, intraoperative decision-making unfolds over seconds to minutes and directly influences organ perfusion and postoperative outcomes. Intraoperative hypotension has consistently been associated with myocardial injury, acute kidney injury and increased mortality, particularly when mean arterial pressure falls below individualized autoregulatory thresholds for sustained periods (16). These associations have made hemodynamic instability a central target for perioperative AI development.

Waveform-based early-warning systems represent one of the most mature intraoperative AI applications. By analyzing high-resolution arterial pressure waveforms and derived features, machine-learning models have been trained to predict hypotension minutes before its onset (6). Randomized controlled trials have demonstrated that, when coupled with predefined hemodynamic treatment protocols, such systems can reduce the cumulative duration and severity of intraoperative hypotension compared with standard care (7, 15). These findings suggest that predictive analytics can modify hemodynamic exposure in real time.

However, predictive accuracy alone is insufficient to establish clinical value. Early-warning systems alter clinician behavior, and therefore function as active interventions. Their evaluation must encompass downstream physiological consequences and behavioral adaptations. Relevant outcomes include time spent within individualized blood pressure targets, cumulative vasopressor dose, total fluid administration, incidence of hypertension, arrhythmias, myocardial injury and acute kidney injury, as well as alert burden and clinician response latency (7, 16). A reduction in hypotension minutes may be offset by increased vasoconstrictor exposure or episodes of reactive hypertension, underscoring the importance of balanced hemodynamic metrics rather than single-threshold endpoints.

Induction and emergence phases introduce additional complexity. During induction, transient hypotension is common and often anticipated, reflecting anesthetic-induced vasodilation and myocardial depression. Predictive systems that do not incorporate phase awareness may generate frequent alerts during this expected transition, potentially prompting excessive vasopressor boluses or unnecessary fluid loading. Such behavior could increase myocardial oxygen demand or precipitate hypertension once sympathetic tone returns. Similarly, during emergence, abrupt changes in ventilation, sympathetic activation and patient movement may degrade signal quality and impair model reliability, increasing false-positive alerts. Incorporating phase-specific logic or contextual awareness into predictive algorithms may mitigate these risks by adapting thresholds and confidence levels according to the stage of anesthesia and surgical context.

Beyond prediction, AI is increasingly explored for assistive and automated control. Reinforcement-learning approaches have been trained on retrospective intraoperative data to recommend vasopressor titration strategies that maintain hemodynamic stability while minimizing drug exposure (8, 19). Closed-loop systems for propofol delivery guided by EEG-derived indices have demonstrated reduced variability in hypnotic depth compared with manual titration (9). Conceptually, such systems shift the role of AI from advisory to semi-autonomous or autonomous control. This transition raises additional safety considerations, including stability under rare physiological conditions, robustness to artifacts and resilience to out-of-distribution inputs.

Dataset shift represents a major challenge in intraoperative AI. Anesthetic practice varies across institutions and countries, including differences in volatile versus total intravenous anesthesia, preferred vasopressors, hemodynamic targets and monitoring density. Policies learned from one environment may encode institutional practice patterns rather than physiological optima, limiting generalizability. Temporal drift is also relevant, as enhanced recovery pathways, new monitoring technologies or evolving pharmacologic strategies alter intraoperative physiology and clinician responses over time. Continuous performance monitoring, periodic recalibration and staged deployment, beginning with silent evaluation, progressing to advisory mode and only then to supervised automation, are therefore critical for safe implementation.

Computer vision adds another intraoperative dimension. In ultrasound-guided regional anesthesia, deep learning models can identify nerves and surrounding structures in real time, facilitating needle alignment and reducing acquisition time, particularly among trainees (24–26). Similar assistive approaches may improve epidural localization or vascular access. In parallel, operating room video analytics can segment instruments and recognize procedural phases, enabling context-aware assistance, workflow optimization and automated documentation. Yet, unlike waveform data, video and high-resolution ultrasound images contain extensive identifiable information about patients and staff. Their integration into AI pipelines necessitates robust consent frameworks, secure storage architectures and explicit governance mechanisms addressing medico-legal responsibility and data reuse (3, 18).

Overall, intraoperative AI moves from prediction to active control within a physiologically unstable and time-sensitive environment. Its safe integration depends not only on predictive performance but also on understanding hemodynamic trade-offs, behavioral responses to alerts, resilience to artifacts and drift, and rigorous governance structures capable of supervising increasingly autonomous systems.

## Postoperative applications

Following surgery, clinical deterioration rarely occurs abruptly. In many cases, subtle physiological deviations, progressive tachycardia, increasing oxygen requirement, rising lactate, declining urine output or altered mental status, precede overt organ failure by several hours. Traditional intermittent monitoring and rule-based early warning scores may fail to detect these evolving patterns early enough to prevent escalation. AI-enhanced ward and ICU surveillance systems attempt to address this limitation by integrating continuous vital signs, laboratory trajectories, medication exposure and perioperative risk profiles into dynamic risk estimates (11–12).

Machine-learning–based early warning systems have demonstrated improved discrimination for unplanned ICU transfer, cardiac arrest and in-hospital mortality compared with conventional scoring systems (12). In perioperative populations, these systems have been associated with earlier activation of rapid response teams and more timely escalation of care, potentially reducing serious adverse events (27). Conceptually, postoperative AI targets the “failure-to-rescue” phenomenon by shortening the interval between physiological deviation and clinical intervention.

However, increasing model sensitivity without proportional gains in specificity risks exacerbating alarm fatigue and workflow disruption. Surgical wards are already saturated with alarms from monitors, infusion pumps and telemetry systems. High false-positive rates may desensitize clinicians and paradoxically delay recognition of true deterioration. Therefore, postoperative AI systems must be evaluated not only for discrimination but also for alert burden, positive predictive value, time-to-intervention, appropriateness of escalations and resource utilization (11, 27). The ultimate metrics of value are reductions in failure-to-rescue, morbidity and mortality, rather than improvements in statistical performance alone.

Integration into existing escalation pathways is equally critical. AI alerts that are not embedded within clear clinical protocols may generate ambiguity rather than action. For example, an elevated deterioration risk score should trigger predefined assessments, laboratory testing, physician review or rapid response activation. Without structured responses, predictive outputs risk remaining informational signals rather than actionable tools. Human factors engineering and co-design with ward staff are therefore central to successful deployment.

Postoperative delirium represents a particularly complex application domain. Delirium is common in older surgical patients and is associated with prolonged hospitalization, functional decline and increased mortality. Machine-learning models incorporating demographic variables, comorbidities, sedative and analgesic exposure, intraoperative hemodynamic patterns and EEG-derived metrics have demonstrated promising discrimination for postoperative delirium (20–21, 28). Observational data link intraoperative EEG suppression and burst suppression to subsequent delirium risk, suggesting a potential mechanistic relationship between depth of anesthesia and postoperative cognitive vulnerability (20–21).

Yet, randomized evidence complicates this interpretation. Trials such as ENGAGES indicate that EEG-guided anesthetic titration alone may not substantially reduce delirium incidence, highlighting the multifactorial nature of the syndrome (29). Delirium risk reflects not only intraoperative neurophysiology but also baseline frailty, inflammation, pain, sleep disruption, infection and medication effects. Predictive models must therefore be situated within comprehensive prevention strategies rather than interpreted as single-threshold triggers.

Methodologically, delirium prediction is challenged by noisy outcome labels and inconsistent assessment tools. Screening frequency, diagnostic criteria and inter-rater variability differ across institutions, introducing measurement bias. Confounding by indication further complicates model interpretation: patients perceived as high risk may receive deeper anesthesia, higher opioid doses or benzodiazepines, which may themselves influence delirium risk. As a result, models may partially encode treatment effects rather than baseline vulnerability.

For delirium prediction to yield clinical benefit, risk stratification must be coupled to explicit, evidence-based prevention bundles including multimodal analgesia, minimization of deliriogenic medications, early mobilization, sleep hygiene and orientation protocols (28–29). Prospective interventional studies are required to demonstrate that model-informed prevention reduces delirium incidence or severity rather than merely improving discrimination.

Explainability tools such as SHAP can support clinical credibility by identifying which variables contribute most strongly to predicted delirium risk (23). In a clinically coherent model, dominant features would include advanced age, baseline cognitive impairment, frailty and benzodiazepine exposure. Excessive reliance on documentation artifacts or post-hoc variables would signal potential information leakage or bias.

In summary, postoperative AI has the potential to reduce failure-to-rescue and improve neurocognitive outcomes, but only if embedded within structured escalation pathways, evaluated for real-world behavioral impact and continuously monitored for unintended consequences. The transition from predictive analytics to effective perioperative intervention remains the central challenge.

## Implementation, workflow-specific risks and algorithmo-vigilance (AI monitoring)

Deployment of perioperative AI requires a fundamental conceptual shift: models must no longer be viewed as static classifiers evaluated once and then implemented, but as dynamic clinical systems embedded within evolving workflows. In perioperative environments characterized by rapid physiological transitions and high-stakes decision-making, AI outputs influence clinician behavior, resource allocation and pharmacologic exposure. As such, these systems should be evaluated with the same methodological rigor applied to drugs or devices.

Model evaluation must therefore extend beyond discrimination metrics such as AUROC. Calibration across clinically relevant risk strata is essential to avoid systematic over- or underestimation of risk. External validation in heterogeneous institutions is critical to ensure generalizability. Decision-curve analysis can quantify net clinical benefit across actionable thresholds, and prospective impact studies, ideally randomized or quasi-experimental, are necessary to demonstrate improved patient outcomes rather than improved statistical performance alone (13–14). Without such evidence, perioperative AI risks remaining an analytic enhancement rather than a therapeutic advance.

Human factors and implementation science are central to this process. Even highly accurate models may fail if they are poorly integrated into electronic workflows, generate excessive alerts or disrupt established team dynamics. Usability testing, clinician feedback loops and iterative threshold tuning are necessary to maintain trust and avoid cognitive overload (15, 27). In high-acuity settings such as the operating room, latency, interface design and alert prioritization directly influence response quality and safety.

In perioperative workflows, AI-related benefits and harms are often tightly coupled. During induction and emergence, for example, hemodynamic variability and artifacts can produce frequent predictive alerts. If these alerts are not phase-aware, clinicians may respond with repeated vasopressor boluses or unnecessary fluid administration, potentially increasing hypertension, arrhythmias or myocardial oxygen demand. Thus, hemodynamic AI systems must be evaluated not only for reduced time in hypotension but also for downstream pharmacologic exposure and adverse physiological trade-offs (16). Balanced hemodynamic endpoints and exposure metrics are essential to avoid replacing one risk with another.

On surgical wards and in ICUs, enhanced surveillance systems may reduce failure-to-rescue by identifying deterioration earlier. However, excessive alert frequency may impair situational awareness and increase workload. Co-design with nursing and medical teams, clearly defined escalation pathways and continuous monitoring of alert burden and response times are required to ensure that predictive systems strengthen rather than fragment clinical vigilance (11). The effectiveness of postoperative AI depends as much on workflow integration as on algorithmic accuracy.

Computer vision applications introduce additional governance considerations. Ultrasound-guided regional anesthesia and operating room video analytics involve high-resolution data streams that contain identifiable patient and staff information. Responsible deployment requires explicit consent processes, secure storage architectures, anonymization procedures and clearly defined policies for data reuse and medico-legal responsibility (3). Transparency regarding data governance is critical for sustaining professional and public trust.

A pervasive challenge across all perioperative AI domains is dataset shift. Changes in case mix, surgical complexity, anesthetic techniques, monitoring density and documentation practices can progressively degrade model performance. Temporal drift may occur silently, without obvious clinical signals, until miscalibration leads to systematic misclassification. Perioperative AI systems therefore require structured algorithmovigilance frameworks inspired by pharmacovigilance (17–18). Such frameworks include continuous monitoring of discrimination and calibration, drift detection algorithms, predefined model update procedures, version control, rollback mechanisms and transparent communication with end users.

Regulatory bodies increasingly recognize that AI-enabled medical software is dynamic and requires lifecycle management. Predefined change-control plans, post-market surveillance and documentation of model updates are becoming expectations rather than optional safeguards. In perioperative contexts, where physiological dynamics are rapid and patient safety margins narrow, robust lifecycle oversight is particularly critical.

Ultimately, perioperative AI should be conceptualized as a sociotechnical system rather than a predictive algorithm. Its safety and effectiveness depend on model performance, workflow design, behavioral responses, governance infrastructure and continuous monitoring. Only by addressing these interdependent dimensions can AI move from promising analytics to reliable clinical practice (30).

## Conclusion

Precision perioperative AI holds substantial promise for improving individualized risk stratification, enhancing hemodynamic stability, supporting procedural accuracy and facilitating earlier detection of postoperative deterioration. Yet predictive capability alone does not ensure clinical benefit. Clear operational definitions, rigorous reference alignment and perioperative-specific evidence are necessary to establish conceptual coherence. Equally important are workflow-aware design, balanced evaluation of benefits and harms, and structured lifecycle monitoring.

By framing perioperative AI as an embedded clinical intervention rather than a static classifier, and by emphasizing calibration, external validation, behavioral impact and algorithmo-vigilance, a pathway emerges for responsible translation into practice. The ultimate benchmark for perioperative AI is not statistical performance but measurable improvements in patient outcomes and safety without introducing new, unintended risks.
