# Supplementary figures and images for: Precision perioperative AI: from signals, images, and records to applications in anesthesia-a narrative mini-review proposing an operational framework
Source: Front Med (Lausanne). 2026 Apr 9;13:1811197. doi: 10.3389/fmed.2026.1811197 (PMC13102806; doi:10.3389/fmed.2026.1811197)

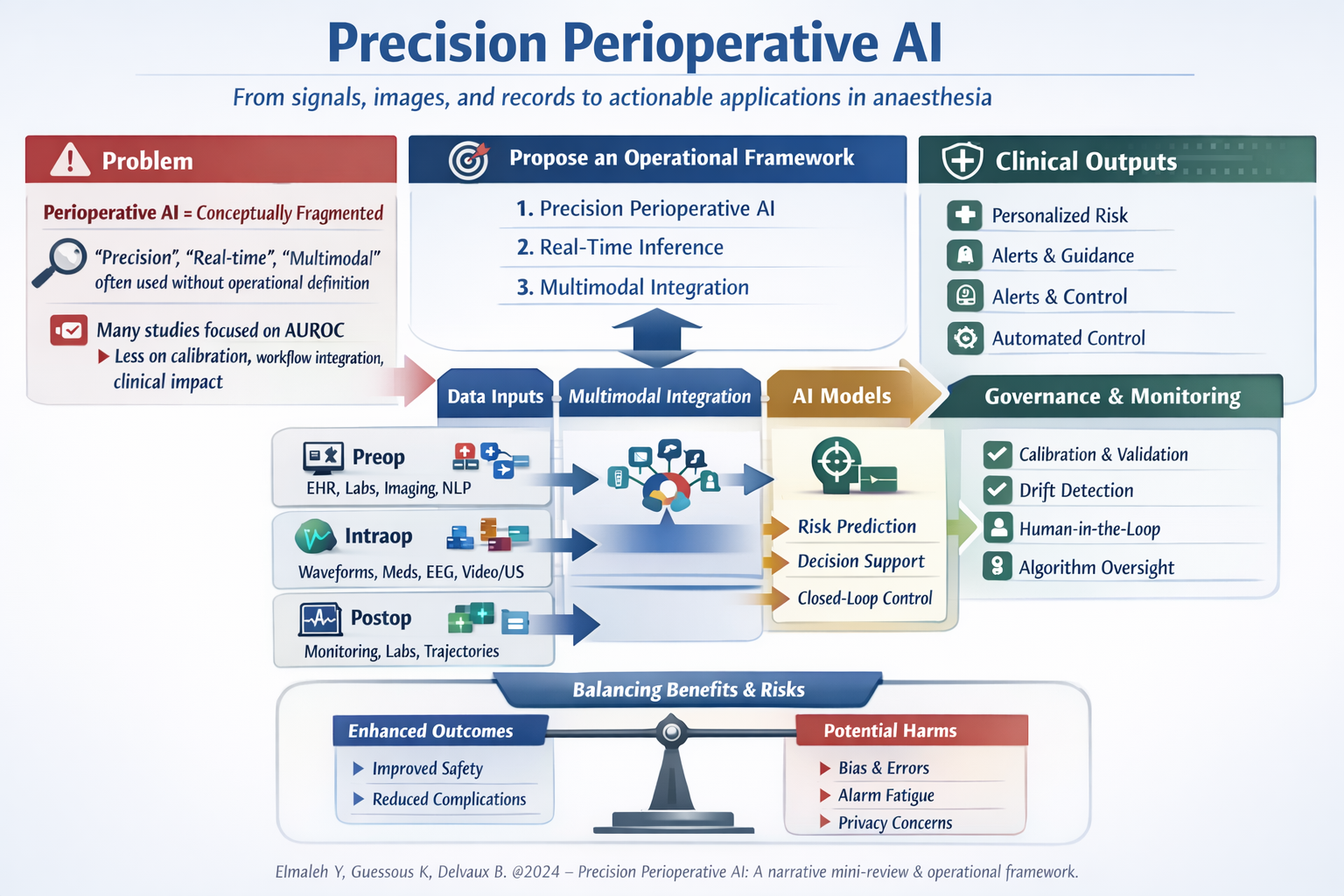

Supplement: Supplementary file 1 [file Image_1.PNG]
